# A trans-diagnostic review of anxiety disorder comorbidity and the impact of multiple exclusion criteria on studying clinical outcomes in anxiety disorders

**DOI:** 10.1038/tp.2016.108

**Published:** 2016-06-28

**Authors:** A N Goldstein-Piekarski, L M Williams, K Humphreys

**Affiliations:** 1 Department of Psychiatry and Behavioral Sciences, Stanford University School of Medicine, Stanford, CA, USA; 2 Department of Sierra-Pacific MIRECC, VA Palo Alto (Sierra-Pacific MIRECC), Palo Alto, CA, USA; 3 VA Center for Innovation to Implementation, Menlo Park, CA, USA

## Abstract

Anxiety disorders are highly comorbid with each other and with other serious mental disorders. As our field progresses, we have the opportunity to pursue treatment study designs that consider these comorbidities. In this perspective review, we first characterized the prevalence of multiple anxiety disorder comorbidity by reanalyzing national survey data, then conducted an English-language PubMed search of studies analyzing the impact of exclusion criteria on treatment outcome data. In the prevalence data, 60% of people with an anxiety disorder had one or more additional anxiety or depression diagnosis. Because our commonly applied exclusion criteria focus on a single diagnosis and do not consider a multiple comorbidity profile, the impact of the criteria may be to exclude up to 92% of anxiety disorder treatment seekers. Moreover, the findings do not suggest a consistent relationship between the number of exclusion criteria and the effect size of treatment outcomes. Thus, future studies might consider a more trans-diagnostic rationale for determining exclusion criteria, one that is generalizable to real-world settings in which multiple diagnoses commonly co-occur. The findings also encourage a more systematic reporting of rationales for the choice of—and the implications of—each exclusion criterion.

## Introduction

Anxiety disorders are the most common class of mental disorders, affecting an estimated 20% of adults (40 million people) in the US.^1, 2, 3, 4^ Of these individuals, at least half experience multiple anxiety disorders and other comorbid conditions such as mood and substance use disorders. Accurate information on the generalizability of treatment outcome studies will therefore depend on having accurate information about how study samples were enrolled and what filters were applied.

The current diagnostic system, DSM-5, distinguishes anxiety disorders (specific phobia (SP), social anxiety disorder (social phobia; SO), panic disorder (PD), agoraphobia and generalized anxiety disorder (GAD)), obsessive-compulsive and related disorders, and trauma- and stressor-related disorders (for example, post-traumatic stress disorder (PTSD)) (see Appendix specific diagnoses). It is presumed that these are independent and discrete disorders, as is major depressive disorder (MDD). Yet, the symptoms often overlap across diagnoses and can vary substantially within diagnoses. The same treatments, spanning pharmacotherapy, behavioral therapy and their combination, are also indicated for the spectrum of anxiety disorders^5^ (Supplementary Table 1). Reflecting these concerns, the Research Domain Criteria (RDoC) initiative is fostering trans-diagnostic research that is explicitly agnostic with respect to filtering by traditional diagnoses to evaluate brain-based mechanisms of dysfunction that may cut across these diagnoses.^6, 7, 8, 9^ Pursuit of RDoC research relies on sample enrollment that is not filtered by diagnosis, but reflects, for example, all people seeking treatment at clinical service.

However, standard practice in treatment outcome studies has been to focus on a particular discrete diagnosis and rule out comorbid disorders. Although exclusion criteria are commonly applied to manage patient safety and confounds with the measurements of interest, exclusions due to comorbidity may limit the relevance of findings for clinical translation. For example, up to ~80% of patients with comorbid conditions are excluded from treatment studies of MDD^10^ and schizophrenia.^11^ A recent study applying typical criteria for clinical trial exclusion to the STAR*D trial showed that these criteria would exclude ~80% of patients seen in primary care, an important issue for prescribers given that that trial findings might not always translate to the patients being treated in usual practice.^12^

An important step towards a complementary RDoC framework for the spectrum of anxiety psychopathology and its comorbidities would be to characterize (i) the extent of comorbidity across anxiety disorders and (ii) the nature and impact of exclusion criteria in anxiety disorder studies of pharmacotherapy, psychotherapy and their combination. To address these issues, this review characterizes the prevalence of anxiety disorders comorbid with each other and with depression by undertaking a secondary analysis of data from the National Comorbidity Survey-Replication (NCS-R).^13, 14^ We then summarize the nature of exclusion criteria used in treatment studies of anxiety disorder; the percentage rates of patients ruled out due to these criteria; and the evidence for the potential impact of these exclusions on the treatment outcome results. Based on the findings, we recommend an approach for developing standardized exclusion criteria reporting relevant to RDoC-motivated and trans-diagnostic research.

## Materials and methods

### Characterizing the prevalence of anxiety disorder comorbidity using national survey data

We undertook a secondary analysis of the NCS-R data to characterize the comorbidity of anxiety disorders with each other and with MDD. The NCS-R survey includes lifetime diagnosis information for 9282 individuals aged ⩾18 years.^13, 14^ DSM-IV diagnosis information was available for five anxiety disorders (PD, GAD, PTSD, SO, and SP) and MDD. See Supplementary Table 2 for the lifetime prevalence of each disorder displayed independently and split by sex. The percentage of lifetime comorbidity across disorders was calculated in several ways. First, to assess the extent of comorbidity across pairs of diagnoses, we determined the proportion of individuals for each diagnostic pairing that had both disorders out of (a) the total number of individuals with at least one anxiety disorder, and (b) the number of individuals diagnosed with each member of the comorbidity pair (for example, out of the individuals with PD those who also had PTSD). Second, a similar set of analyses was conducted looking at diagnoses triplets (for example, number of individuals who were comorbid for GAD, as well as PTSD and PD). The percentages of comorbidity were then represented as heat maps (created in R; R Foundation for Statistical Computing, Vienna, Austria; URL http://www.R-project.org)^15^ with darker colors indicating higher percentage of comorbidity.

### The nature and impact of exclusion criteria based on a meta-review of studies

We then completed a structured literature review of the impact of exclusion criteria on anxiety disorder treatment outcome research. This review was part of the Cross-Disease Review of Exclusion Across Medicine (CREAM) project, a structured literature review of studies of exclusion criteria and their impact across a range of medical specialties (for example, oncology, cardiology, rheumatology and psychiatry).

### Search strategy and selection criteria

In the Cross-Disease Review of Exclusion Across Medicine (CREAM) project, literature was identified systematically by conducting English-language searches in PubMed (Date of Search: Oct 1, 2014 on the following terms: “Eligibility criteria and generalizability” (anywhere in paper), “exclusion criteria and generalizability” (anywhere in paper), “exclusion criteria” (in title of paper) and “eligibility criteria” (in title of paper). To be considered relevant, studies had to analyze data on i) the prevalence and nature of exclusion criteria, ii) the overall and specific rates of exclusion due to commonly used exclusion criteria, and/or iii) the impact of exclusion criteria on sample representativeness or outcomes. From this cross-disease pool of literature, evidence on individual diseases was extracted for focused reviews, in this case studies addressing anxiety disorders.^11^

## Results

### The prevalence of anxiety disorder comorbidity

**Figure 1:**
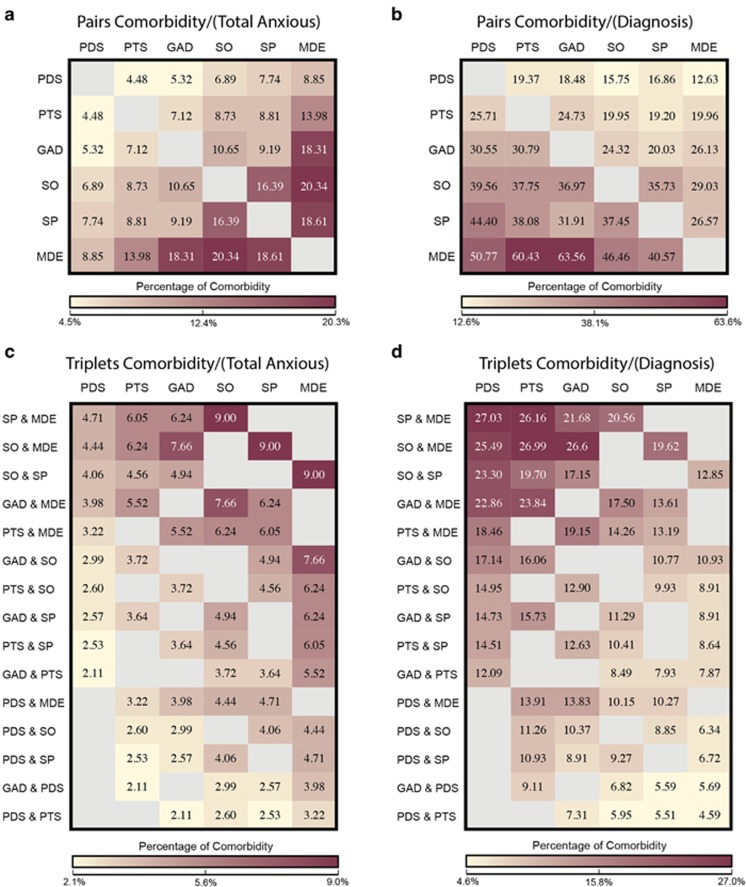
Proportion of lifetime comorbidity heat maps. Proportion of individuals with (**a**) pairs of diagnoses out of those who had at least one anxiety disorder diagnosis (*N=*2611); (**b**) pairs of diagnoses using the disorder listed in the column header as the denominator; (**c**) three diagnoses out of those who had at least one anxiety disorder diagnosis and; (**d**) three diagnoses using the disorder listed in the column header as the denominator. For example, the top left cell represents the proportion of individuals with panic disorder who also had specific phobia and major depressive disorder. These results were generated from a secondary analysis of the National Comorbidity Study data.^13, 14^ GAD, generalized anxiety disorder; MDD, major depressive disorder; PD, panic disorder; PTSD, post-traumatic stress disorder; SO, social phobia; SP, specific phobia.

Sixty percent of individuals with one anxiety disorder had at least one other anxiety disorder or depression diagnosis, and 27% of individuals had three or more of these disorders comorbid. Importantly, comorbidity rates differed across disorders (Figure 1). For diagnostic pairs, the proportion of individuals with two comorbidities ranged from 4.5 to 20.3% within those diagnosed with at least one anxiety disorder (Figure 1a). The greatest comorbidity rates were observed between pairs of SO-MDD (20.3%), MDD-SP (18.6%) and MDD-GAD (18.3%), and the lowest comorbidity rates between pairs of PD-PTSD (4.5%) and PD-GAD (5.3%). These percentages differed when looking at comorbidity profiles within each diagnosis separately (for example, percentage of individuals with PD who also had GAD, PTSD, SO, SP, or MDD), such that the proportion of individuals with a given disorder who also had a second disorder (for example, individuals with MDD who also had PTSD) was not the same as the proportion of individuals with the second disorder who also had the first disorder (for example, individuals with PTSD who also had MDD) (Figure 1b). Notably, individuals diagnosed with either PTSD or GAD had a high percentage of comorbidity with MDD (60.4% and 63.6%, respectively); however, those with MDD had a relatively lower percentage of comorbidity with PTSD or GAD (20.0% and 26.1%, respectively).

Similar findings were also observed when considering comorbidity triplets (Figures 1c and d).

### The nature and impact of exclusion criteria in anxiety disorder treatment research

The papers returned from the search results can be categorized roughly into three types: those that (i) reviewed the prevalence of exclusion criteria in treatment research studies, (ii) reviewed the rates of exclusion by applying commonly used exclusion criteria to an independent sample of treatment-seeking individuals, and (iii) evaluated the impact of exclusion criteria on outcomes or sample representativeness. Table 1 gives a specific breakdown of the studies that covered each of these topics, organized according to the specific anxiety disorder of focus in each study.

#### Prevalence of exclusion criteria

Eight publications provided information regarding the prevalence of exclusion criteria across studies of PTSD, PD, GAD, SO and obsessive-compulsive disorder (OCD)^16, 17, 18, 19, 20, 21, 22, 23^ (Table 2; further details in Supplementary Results). However, one study that reported commonly used exclusion criteria for GAD did not report the actual usage frequency of independent exclusion criteria.^21^ Studies varied greatly in how they explained exclusion criteria. Search dates are not readily available since some studies used other meta-analyses. Some studies only listed the most common exclusion criteria and did not include actual frequency of use. These studies are summarized and described in Table 2.

Together, these findings indicate that while numbers of exclusion criteria vary across empirical studies and disorders, several are used in greater frequency than others. The most commonly reported exclusion criteria in studies across disorders were psychosis (80% of all studies), substance dependence (69% most were even more expansive excluding for any substance use/misuse: 41%), bipolar disorder (47%), comorbid MDD (39%) and suicide risk (36%).

Psychotherapy randomized controlled trials (RCT) have slightly fewer exclusion criteria than pharmacotherapies. This is apparent for both GAD and SO, for which the median numbers of exclusion criteria for psychotherapy were 5 and 7, respectively, whereas the median numbers for pharmacotherapy were 11 and 10, respectively.

#### Rates of exclusion

Nine publications provided information regarding how many patients are being excluded as a result of commonly applied exclusion criteria, reporting on exclusion rates across PTSD, OCD, PD, GAD and SO.^16, 18, 19, 20, 21, 23, 24, 25, 26^ Of these, four categorized the exclusion rates of specific exclusions by first performing a meta-analysis to determine commonly used exclusion criteria and then applying these exclusion criteria to an independent database of treatment-seeking individuals for OCD, PD, GAD and SO^19, 20, 21, 23^ (Table 3). Three reviewed the exclusion rates by aggregating percentages of exclusion across previously published studies.^16, 18, 26^ The remaining two presented exclusion rates from individual empirical studies assessing the impact of exclusion criteria on treatment efficacy.^24, 25^

Combining the findings from these studies yielded several clinically relevant observations regarding exclusion criteria rates. First, the percentage of individuals that would have been excluded due to at least one exclusion criteria was substantial, ranging from 72% of those with OCD^19^ up to as much as 92.4% of those with PD.^20^ Second, not surprisingly, the percentage of individuals who would have been excluded varied dramatically by the type of exclusion and the primary disorder studied. For example, the percentage of participants excluded due to a psychosis diagnosis was relatively small yet ranged from 2.8% of those with OCD^19^ to 21.2% of those with PD.^20^ Similarly, the percentage of those excluded due to comorbid depression varied from 16.3% of those with OCD to 70.6% of those with a PD diagnosis.

Further findings regarding the general and specific exclusion rates due to application of exclusion criteria are presented in more detail for each study across anxiety diagnoses in the Supplementary Results.

#### Impact of exclusion criteria on sample representativeness and outcomes

Seven publications provided information regarding the impact of exclusion criteria on sample representativeness and outcomes across PTSD, OCD, PD, GAD and SO.^16, 22, 24, 25, 26, 27, 28^ Of these, four were meta-analyses focused mostly on clinical outcomes that compared effect sizes across studies that included varying degrees of exclusion criteria.^16, 22, 26, 28^ The remaining three compared demographics and outcomes between patients who would have been excluded due to commonly used exclusion criteria and those who would have been included.^25, 27, 29^ One meta-analysis^26^—which collapsed across PD, GAD, and depression and was based on eight studies—reported a negative correlation between the number of exclusion criteria described in the methods section of each article and the percentage of treatment seekers at termination. Given the rather large differences in methodology and conclusions of the above studies, the impact of exclusion criteria on sample representativeness and outcomes are provided below for each diagnosis separately.

PTSD impact on representativeness: No study to date has examined differences in demographics or baseline clinical symptoms between patients typically excluded from RCTs and those included.

PTSD impact on clinical outcome: To determine the impact of exclusion criteria on clinical trial outcomes, Bradley *et al.*^16^ correlated the number of exclusion criteria from each of the 26 studies in their meta-analysis with the pre- versus post-treatment effects sizes. Number of exclusion criteria was significantly related to pre- versus post-treatment effect size (*r*=0.42, df=23, *P=*0.03), such that studies with more exclusion criteria reported higher effect sizes.

OCD impact on representativeness: In 2000, Franklin *et al.*^24^ compared demographic and outcome information from a group of 110 individuals who had either been excluded from (79%) or refused to participate in (21%) RCTs conducted at the Center for the Treatment and Study of Anxiety (CTSA) to that of individuals from four previously conducted RCTs. Although they did not separate the demographic information for refusers and excluded individuals, demographic information on mean ages (CTSA sample=34.2, RCT samples=34.8, 33.8, 30.5 and 31.6), gender ratios (CTSA=47% females, RCT=46%, 53%, 55%, 56% female) and education (CTSA=45% with undergraduate or graduate education, RCT=34 and 44%) were similar across studies. Many pieces of the demographic information for the previously conducted RCTs were not available for several or all of the included studies. Pretreatment OCD severity was comparable between samples.

OCD impact on clinical outcome: Franklin *et al.*^24^ found that change in pre- to post-treatment severity for OCD symptoms in their CTSA study was similar to two previously conducted RCTs, but greater than two others (CTSA mean reduction=60%, RCT samples reduction=62, 54, 40 and 32%). Mirroring the reduction in OCD symptoms, they also found that depression severity—as indexed by Beck Depression Inventory (BDI) scores—also improved to a greater extent than in the three available RCTs (CTSA mean reduction=57%, RCTs sample reduction=39%, 43%, and an increase in one of the RCTs of 15%). To further examine the effects of treatment across the samples, they calculated effect sizes from the change in pre- versus post-treatment OCD and depression severity. Regarding the OCD severity, the effect size for the CTSA sample was larger than three out of the four RCT samples, and slightly smaller than one sample (CTSA effect size=3.26, RCT effect sizes=2.31, 0.93, 3.88 and 1.00). The CTSA sample also had a larger effect size for the pre- to post-treatment change in depression severity (CTSA effect size=1.26, RCT effect sizes=0.93, 0.79 and −0.33).

Panic disorder impact on representativeness: In 2002, Mavissakalian and Guo^25^ examined the differences in demographic variables of patients who were excluded from a drug trial for OCD and those who were accepted. Those who were excluded were more likely to be male (88 vs 77%), have a later OCD onset (30.6 vs 26.8 years) and a shorter duration of illness (7.3 vs 10.0 years). However, a potential explanation for the differences in duration of illness and onset of OCD was that exclusion criteria for this study included a minimum number of panic attacks per month and at least moderate PD severity. Overall, likely as a consequence of these restrictions, the accepted group had significantly lower functioning and higher anxiety scores than the excluded group.

GAD: No study to date has examined differences in demographics or baseline clinical symptoms between patients typically excluded from RCTs and those included.

SO impact on representativeness: Juster *et al.*^27^ conducted an empirical study comparing demographic information and clinical outcomes of participants who were entered into an RCT (*n=*47) to those deemed ineligible to participate in the drug arm (*n=*28). Both groups received cognitive behavioral therapy (CBT) for SO. No differences were found between accepted and excluded patients regarding gender ratio, age, employment, education or number of years with SO. Similarly, accepted participants did not differ from those excluded regarding 8 questionnaires or 10 independent assessor measures of severity.

SO impact on clinical outcome: In terms of outcomes, those enrolled were significantly different than those excluded on only three measures of the 8 questionnaires and 10 independent assessor measures of severity.^27^ Specifically, enrolled participants showed a significantly larger improvement in global functioning, social anxiety and avoidance.

In 2003, Lincoln *et al.*^28^ calculated pre- to post-treatment effect sizes for subgroups characterized by exclusion criteria in a sample of 217 patients who received exposure therapy combined with cognitive restructuring treatment for SO. Common exclusion criteria included (1) comorbid depression (as measured by a BDI ⩾18), (2) prior psychological treatment for SO, (3) low symptom severity, (4) >50 or <20 years of age and (5) comorbid Axis I disorder. The effect size for the sample that excluded individuals with higher depression (effect size=0.76) was smaller than for the sample that included individuals with high levels of depression (effect size=1.28).

A second study by Lincoln *et al.*^22^ calculated effect sizes for the change in pre- to post-treatment symptom severity for 26 clinical trial investigations of CBT and exposure therapies for SO conducted between 1996 and 2002. They next categorized studies based on their usage of exclusion criteria and created averaged effect sizes for each category (studies were weighted with the square root of *n*). Exclusion criteria included in this investigation were (1) comorbid psychosis, substance misuse or bipolar disorder, (2) comorbid depression, (3) comorbid Axis 1 disorder, (4) comorbid antisocial personality disorder, (5) low symptom severity and (6) prior treatment. The difference in effect sizes between two groups of studies were considered meaningful if the weighted means differed by at least 0.3. The only meaningful difference was between those studies that excluded prior treatment (effect size=0.71) and those that allowed prior treatment (effect size=1.01). Although slightly below their threshold, the effect size increased from 0.77 to 0.94 when excluding comorbid psychosis, substance misuse or bipolar disorder.

In both studies by Lincoln *et al.*,^22, 28^ an accumulation of common exclusion criteria was not generally found to lead to higher effect sizes.

## Discussion

Our review of exclusion criteria used in trials of anxiety disorders shows that restricting the study sample to a discrete homogenous diagnosis with limited comorbidities may exclude up to 92% of treatment seekers. As a consequence, existing trials may have limited applicability for guiding future RDoC-motivated trans-diagnostic studies of treatment mechanisms or for translation to real-world settings, where comorbidity is the norm rather than the exception. A step towards addressing these issues would be for authors to adopt a standardized way of reporting the rationale for all exclusion criteria and provide data on the likely translational impact of these criteria.

### Multiple comorbidities

Comorbidity of anxiety disorders with each other and with mood disorders is the most immediate issue to consider in regard to the impact of exclusion criteria. Current comorbidity data tend to focus on pair-wise combinations of anxiety and mood disorders, and there are no hard numbers about the prevalence of multiple combinations of anxiety disorders. These numbers are important for guiding new approaches to defining trans-diagnostic samples and RDoC-motivated samples unfiltered by a discrete diagnosis. Our initial analysis of lifetime comorbidity survey data showed that at least 60% of participants with one anxiety disorder had one or more additional anxiety or depression diagnosis. Further we show that the pattern of lifetime comorbidity is not bi-directional. For example, 60% of those with PTSD also had a diagnosis of MDD, however, only 20% of those diagnosed with MDD additionally had a PTSD diagnosis. Similarly, when considering comorbidity triplets 26% of those with a lifetime diagnosis of PTSD also had a diagnosis of MDD and social phobia, where only 9% of those with MDD had an additional PTSD and social phobia diagnosis. These patterns suggest that use of exclusion criteria may be disproportionally impacting certain anxiety diagnoses, such as PTSD, more than others.

### The impact of exclusion for comorbidity

Given that the same treatments are currently used for the spectrum of anxiety disorders, the rationale for excluding participants due to a second or third additional anxiety disorder and/or MDD is unclear. Studies that followed the excluded individuals found mixed pattern of effects, from no differences to both better and worse outcomes. Thus, the impact of additional anxiety disorders is unlikely to consistently produce heterogeneity that confounds the results or reduces statistical power. Pragmatically, recruitment periods might be shortened by the opportunity to enroll all treatment seekers meeting anxiety disorder criteria for one or more disorders. Our findings suggest that in future anxiety trials, a cross-cutting RDoC approach in which participants present all forms of anxiety disorder that occur within a real-world setting may be of value for informing clinical care. This would require a fresh approach to trial design to record heterogeneous clinical profiles that reflect population data^1^ rather than attempt to define *a priori* specific diagnostic boundaries.

Our review suggests that, because of their common co-occurence, excluding for multiple other anxiety and mood disorders in order to study a 'single' anxiety disorder has the greatest potential for impacting the interpretation of treatment outcomes. Other commonly applied exclusion criteria do not necessarily have a big impact, feasibly reflecting a lower frequency of comorbidity. Of the studies we reviewed, the most common exclusion criterion was psychosis. However, this criterion typically rules out a comparatively small number of potential participants (2.8–21.2%), reflecting a lower frequency of those with anxiety who also have psychosis and may therefore have less impact on results than other criteria. In some studies, it might also be relevant to include psychosis as a comorbid clinical feature relevant to the treatment of interest. For example, patients with severe comorbid disorders such as psychosis may still benefit from psychotherapy treatments such as CBT used to treat PTSD (see refs 17, 30). In contrast, depression comorbidity produced the highest rates of exclusion, even though these rates were also variable (8.7–70.6%).

### The need for standardized reporting of exclusion criteria

Overall, our review highlighted the paucity of evidence concerning the nature and impact of exclusion criteria for anxiety disorder treatment studies. Because significant variation exists in the specific set of exclusion criteria used in each trial, the generalizability of the results is limited. Variation in the number and type of exclusion criteria used, and in specific cutoff scores on psychometric scales, can also make it difficult to compare the results of studies of the same treatment. Exclusion criteria tend to appear as if they are 'cut and pasted' into the methods section, and may not be systematically reported or may even be underreported.^31, 32^ Our findings encourage the systematic reporting of the rationale used to guide the choice of each exclusion criteria and the implications of the choice. A recommendation based on the current review is that for all exclusion criteria, manuscripts should report the criteria in a specific manner for it to be replicable, present the rationale for each criterion and provide data on its impact. The justification and rationale could include information on the choice of each criterion and focus on criteria that reflect the clinical purpose of the study (for example, to gather data relevant to routine clinical care, impose only exclusion criteria that are medically necessary or that a reasonable clinician in practice would impose). To consider the impact of exclusion criteria, authors might include data on the proportion of subjects ruled out by the criterion and whether anything is known about disproportionate impact of the criterion (for example, disproportionately excluded female or racial/ethnic minority subjects). A first step towards achieving such standardized reporting of exclusion criteria could be for authors to use the CONSORT guidelines, which require a thorough reporting of exclusion criteria for each trial, and for journals to adopt these guidelines.

### Limitations of the current state of knowledge

Due to the dearth of current evidence (that is, only 13 reviews/meta-analyses to date), the current state of knowledge is not based on a systematic study of the topic. Specific limitations of the existing knowledge base include the lack of clarity regarding screening methods and the lack of information about comorbidity.^16^ Of course, these limitations reflect the inherent limitations of clinical trials that have tended to not include the details about exclusion criteria (or the rationale for exclusion). As a result, reviews of exclusion criteria have focused on a limited subset of anxiety disorders, the description of exclusion criteria is commonly vague (for example, 'major mental illness') and there is a lack of consistency in criteria due to the patchy rate of review (eight reviews prior to 2004 and five since) spanning three revisions to the DSM.

To address these limitations, systematic protocols must be developed for studying the impact of exclusion criteria.

## Figures and Tables

**Table 1:** Summary of the studies reviewed

	*Studies organized according to the disorders of focus in each study*				
*Topics covered in the review*	*PTSD*	*OCD*	*PD*	*GAD*	*SO*
Reviewed prevalence of exclusion criteria across studies	Bradley *et al.*^16^ Ronconi *et al.*^17^	Eddy *et al.*^18^ Odlaug *et al.*^19^	Hoertel *et al.*^20^	Hoertel *et al.*^21^	Lincoln and Reif^22^ Hoertel *et al.*^23^
Applied exclusion criteria to other database	—	Odlaug *et al.*^19^	Hoertel *et al.*^20^	Hoertel *et al.*^21^	Hoertel *et al.*^23^
Presented extent of exclusions from single study	—	Franklin *et al.*^24^	Mavissakalian and Guo^25^	—	—
Presented extent of exclusions across many studies	Bradley *et al.*^16^	Eddy *et al.*^18^	Westen and Morrison^26^	Westen and Morrison^26^	—
Impact of exclusion criteria on sample representativeness	—	Franklin *et al.*^24^	Mavissakalian and Guo^25^	—	Juster *et al.*^27^
Impact of exclusion criteria on outcomes	Bradley *et al.*^16^	Franklin *et al.*^24^	Westen and Morrison^26^	Westen and Morrison^26^	Juster *et al.*^27^ Lincoln and Reif^22^ Lincoln *et al.*^28^

Abbreviations: GAD, generalized anxiety disorder; OCD, obsessive-compulsive disorder; PD, panic disorder; PTSD, post-traumatic stress disorder; SO, social phobia.

**Table 2:** Summary of the prevalence of exclusion criteria applied in treatment studies of anxiety disorders

*Review paper*	*Bradley* *et al.*^16^	*Ronconi* *et al.*^17^	*Eddy* *et al.*^18^	*Eddy* *et al.*^18^	*Hoertel* *et al.*^20^	*Lincoln and Reif*^22^
*Reviews of the reported prevalence of exclusion criteria*						
Year range of studies	1980–2003	1980–2012	1980–2001	1980–2001	1980–2004	1996–2002
Disorder	PTSD	PTSD	OCD	OCD	PD	SO
Type of treatment	Psychotherapy	Psychotherapy	Psychotherapy	Pharmacotherapy	Pharmaco/psychotherapy	CBT/exposure
Number of studies reviewed	26	75	15	32	20	26^a^
						
*Percentage prevalence (and number) of studies excluding patients according to each exclusion criterion*						
* Other psychiatric disorders*						
* *Any DSM Axis I disorders				31.2% (10)		38.5% (10)
* *MDD		28.0% (21)	20.0% (3)	59.4% (19)	75.0% (15)	61.5% (16)
* *Suicide risk	46.2% (12)	58.7% (44)	20.0% (3)	18.8% (6)	25.0% (5)	
* *Bipolar disorder		58.7% (44)		56.3% (18)	35.0% (7)	88.5% (23)
* *Psychosis	84.6% (22)	90.7% (68)	66.7% (10)	68.8% (22)	80.0% (16)	88.5% (23)
* *Any DSM Axis II disorders			6.7% (1)	15.6% (5)	15.0% (3)	
Avoidant personality disorder			6.7% (1)	15.6% (5)	15.0% (3)	26.9% (7)
Antisocial personality disorder						
						
* Alcohol abuse/dependence*						
Drug or alcohol abuse/dependence	61.5% (16)					
* *Substance^b^ use			46.7% (7)	68.8% (22)		88.5% (23)
* *Substance^b^ abuse	61.5% (16)	44.0% (33)	46.7% (7)	68.8% (22)	60.0% (12)	88.5% (23)
* *Substance^b^ dependence	61.5% (16)	72.0% (54)	46.7% (7)	68.8% (22)	60.0% (12)	88.5% (23)
						
* Treatments*						
* * Current psychotherapy			60.0% (9)	68.8% (22)	20.0% (4)	
* *Recent medication changes		41.3% (31)				
* * Current pharmacotherapy			60.0% (9)	68.8% (22)	35.0% (7)	
* * History of treatment			26.7% (4)			20.7% (6/29)
						
* Other specific anxiety disorders*						
* * Panic disorder				50.0% (16)		38.5% (10)
* *Agoraphobia				43.8% (14)		38.5% (10)
* * Eating disorder				43.8% (14)		38.5% (10)
* * OCD					30.0% (6)	38.5% (10)
Specific & social phobias						
						
* Other medical conditions*						
* * Major medical condition			20.0% (3)	71.9% (23)	55.0% (11)	
* *Organic disorder	76.9% (20)		66.7% (10)	68.8% (22)	35.0% (7)	
* *Serious comorbidity^c^	61.5% (16)					
* *Exclusion of low severity						43.3% (13/30)
* * Pregnancy					25.0% (5)	

Abbreviations: CBT, cognitive behavioral therapy; DSM, Diagnostic and Statistical Manual of Mental Disorders; MDD, major depressive disorder; OCD, obsessive-compulsive disorder; PD, panic disorder; PTSD, post-traumatic stress disorder; SO, social phobia.

a Four additional studies did not provide information regarding the most common exclusion criteria.

b Substance use/abuse/dependence includes both alcohol and/or drug use/abuse/dependence.

c General term, Bradley *et al.*^16^ notes that the definition of serious comorbidity was often not described.

**Table 3:** Summary of the rates of exclusion because of each exclusion criterion applied in in treatment studies of anxiety disorders

	*Reviews of rates of exclusion*					
*Review paper*	*Odlaug*^19^	*Hoertel*^20^	*Hoertel*^21^	*Hoertel*^21^	*Hoertel*^23^	*Hoertel*^23^
Year range of studies reviewed	1980–2010	1898–2004	1980–2009	1980–2009	1979–2007	1979–2007
Disorder	OCD	PD	GAD	GAD	SO	SO
Type of treatment	Pharmacotherapy	Pharmacotherapy and psychotherapy	Pharmacotherapy	Psychotherapy	Pharmacotherapy	Psychotherapy
Number of participants	325	105^a^	329^a^	329^a^	363^a^	363^a^
						
*Exclusion criteria*	*Rates of exclusion reported in each review according to each exclusion criterion*					
*Other psychiatric disorders*						
Axis I disorder						
MDD	16.3%	70.6%	62.8%	62.8%	60.4%	60.4%
Suicide risk					5.9%	
Bipolar disorder	3.1%	58.9%	20.0%		42.6%	42.6%
Psychosis	2.8%	21.2%	5.9%	5.9%	14.5%	14.5%
Axis II disorders						
Avoidant personality disorder						
Antisocial personality disorder			10.4%			
						
*Alcohol abuse/dependence*						
Alcohol abuse/dependence	5.2%	61.5%	12.9%		16.4%	16.4%
Drug abuse/dependence	5.2%	61.5%	5.7%		8.7%	8.7%
Substance abuse/dependence	5.2%	61.5%				
						
*Treatments*						
Current psychotherapy						
Recent medication changes						
Current pharmacotherapy						
History of treatment						
						
*Other specific anxiety disorders*						
Panic disorder				36.4%	37.6%	
Agoraphobia						
Eating disorder						
OCD						
Specific and social phobia				47.5%		
						
*Other medical conditions*						
Major medical condition		34.3%	19.4%		25.3%	
Organic disorder						
Serious comorbidity						
Exclusion of low severity	28.9%					
Pregnancy			3.1%		2.2%	
% Excluded for at least 1 exclusion criteria	72.0%	92.4%	81.8%	82.1%	87.8%	80.5%

Abbreviations: GAD, generalized anxiety disorder; MDD, major depressive disorder; OCD, obsessive-compulsive disorder; PD, panic disorder; SO, social phobia.

a Percentages are out of the treatment-seeking sample.

## APPENDIX

### Anxiety disorders

Separation anxiety disorder

Selective mutism

Specific phobia

Social anxiety disorder (social phobia)

Panic disorder

Panic attack (specifier)

Agoraphobia

Generalized anxiety disorder

Substance/medication-induced anxiety disorder

Anxiety disorder due to another medical condition

Other specified anxiety disorder

Unspecified anxiety disorder

### Obsessive-compulsive and related disorders

Obsessive-compulsive disorder

Body dysmorphic disorder

Hoarding disorder

Trichotillomania (hair-pulling disorder)

Excoriation (skin-picking) disorder

Substance/medication-induced obsessive-compulsive and related disorder

Obsessive-compulsive and related disorder due to another medical condition

Other specified obsessive-compulsive and related disorder

Unspecified obsessive-compulsive and related disorder

### Trauma- and stressor-related disorders

Reactive attachment disorder

Disinhibited social engagement disorder

Posttraumatic stress disorder

Acute stress disorder

Adjustment disorders

Other specified trauma- and stressor-related disorder

Unspecified trauma- and stressor-related disorder

## References

[bib1] Kessler RC, Berglund P, Demler O, Jin R, Merikangas KR, Walters EE. Lifetime prevalence and age-of-onset distributions of DSM-IV disorders in the National Comorbidity Survey Replication. Arch Gen Psychiatry 2005; 62: 593–602. 15939837 10.1001/archpsyc.62.6.593

[bib2] Substance Abuse and Mental Health Services AdministrationResults from the 2012 National Survey on Drug Use and Health: Mental Health Findings. Rockville, MD, USA, 2013.

[bib3] Somers JM, Goldner EM, Waraich P, Hsu L. Prevalence and incidence studies of anxiety disorders: a systematic review of the literature. Can J Psychiatry 2006; 51: 100–113. 16989109 10.1177/070674370605100206

[bib4] Weissman MM, Bland RC, Canino GJ, Faravelli C, Greenwald S, Hwu HG et al. The cross-national epidemiology of panic disorder. Arch Gen Psychiatry 1997; 54: 305–309. 9107146 10.1001/archpsyc.1997.01830160021003

[bib5] Cuijpers P, Sijbrandij M, Koole SL, Andersson G, Beekman AT, Reynolds CF3rd. The efficacy of psychotherapy and pharmacotherapy in treating depressive and anxiety disorders: a meta-analysis of direct comparisons. World Psychiatry 2013; 12: 137–148. 23737423 10.1002/wps.20038 PMC3683266

[bib6] Insel T, Cuthbert B, Garvey M, Heinssen R, Pine DS, Quinn K et al. Research domain criteria (RDoC): toward a new classification framework for research on mental disorders. Am J Psychiatry 2010; 167: 748–751. 20595427 10.1176/appi.ajp.2010.09091379

[bib7] Cuthbert BN. The RDoC framework: facilitating transition from ICD/DSM to dimensional approaches that integrate neuroscience and psychopathology. World Psychiatry 2014; 13: 28–35. 24497240 10.1002/wps.20087 PMC3918011

[bib8] Cuthbert BN, Insel TR. Toward the future of psychiatric diagnosis: the seven pillars of RDoC. BMC Med 2013; 11: 126. 23672542 10.1186/1741-7015-11-126 PMC3653747

[bib9] Sorel E. 21st Century Global Mental Health. Jones & Bartlett Learning: Boston, MA, USA, 2013.

[bib10] Halvorson MA, Humphreys K. A review of the nature and impact of exclusion criteria in depression treatment outcome research. Ann Depress Anxiety 2015; 2: 1058.

[bib11] Humphreys K. A review of the impact of exclusion criteria on the generalizability of schizophrenia treatment research. Clin Schizophr Relat Diagn 2014; 20: 1–25. 28548580

[bib12] Preskorn SH, Macaluso M, Trivedi M. How commonly used inclusion and exclusion criteria in antidepressant registration trials affect study enrollment. J Psychiatr Pract 2015; 21: 267–274. 26164052 10.1097/PRA.0000000000000082

[bib13] Kessler RC, Berglund P, Chiu WT, Demler O, Heeringa S, Hiripi E et al. The US National Comorbidity Survey Replication (NCS-R): design and field procedures. Int J Methods Psychiatr Res 2004; 13: 69–92. 15297905 10.1002/mpr.167 PMC6878537

[bib14] Kessler RC, Merikangas KR. The National Comorbidity Survey Replication (NCS-R): background and aims. Int J Methods Psychiatr Res 2004; 13: 60–68. 15297904 10.1002/mpr.166 PMC6878416

[bib15] R Development Core Team. R: A language and environment for statistical computing. R Foundation for Statistical Computing, Vienna, Austria, 2008. ISBN 3-900051-07-0. Available at: http://www.R-project.org.s.

[bib16] Bradley R, Greene J, Russ E, Dutra L, Westen D. A multidimensional meta-analysis of psychotherapy for PTSD. Am J Psychiatry 2005; 162: 214–227. 15677582 10.1176/appi.ajp.162.2.214

[bib17] Ronconi JM, Shiner B, Watts BV. Inclusion and exclusion criteria in randomized controlled trials of psychotherapy for PTSD. J Psychiatr Pract 2014; 20: 25–37. 24419308 10.1097/01.pra.0000442936.23457.5b

[bib18] Eddy KT, Dutra L, Bradley R, Westen D. A multidimensional meta-analysis of psychotherapy and pharmacotherapy for obsessive-compulsive disorder. Clin Psychol Rev 2004; 24: 1011–1030. 15533282 10.1016/j.cpr.2004.08.004

[bib19] Odlaug BL, Weinhandl E, Mancebo MC, Mortensen EL, Eisen JL, Rasmussen SA et al. Excluding the typical patient: thirty years of pharmacotherapy efficacy trials for obsessive-compulsive disorder. Ann Clin Psychiatry 2014; 26: 39–46. 24501729 PMC4236296

[bib20] Hoertel N, Le Strat Y, De Maricourt P, Limosin F, Dubertret C. Are subjects in treatment trials of panic disorder representative of patients in routine clinical practice? Results from a national sample. J Affect Disord 2013; 146: 383–389. 23084184 10.1016/j.jad.2012.09.023

[bib21] Hoertel N, Le Strat Y, Blanco C, Lavaud P, Dubertret C. Generalizability of clinical trial results for generalized anxiety disorder to community samples. Depress Anxiety 2012; 29: 614–620. 22495990 10.1002/da.21937

[bib22] Lincoln TM, Rief W. How much do sample characteristics affect the effect size? An investigation of studies testing the treatment effects for social phobia. J Anxiety Disord 2004; 18: 515–529. 15149711 10.1016/S0887-6185(03)00040-9

[bib23] Hoertel N, de Maricourt P, Katz J, Doukhan R, Lavaud P, Peyre H et al. Are participants in pharmacological and psychotherapy treatment trials for social anxiety disorder representative of patients in real-life settings? J Clin Psychopharmacol 2014; 34: 697–703. 25154011 10.1097/JCP.0000000000000204

[bib24] Franklin ME, Abramowitz JS, Kozak MJ, Levitt JT, Foa EB. Effectiveness of exposure and ritual prevention for obsessive-compulsive disorder: randomized compared with nonrandomized samples. J Consult Clin Psychol 2000; 68: 594–602. 10965635

[bib25] Mavissakalian MR, Guo S. Predictors of entering a long-term drug treatment study of panic disorder. Compr Psychiatry 2002; 43: 88–94. 11893985 10.1053/comp.2002.30803

[bib26] Westen D, Morrison K. A multidimensional meta-analysis of treatments for depression, panic, and generalized anxiety disorder: an empirical examination of the status of empirically supported therapies. J Consult Clin Psychol 2001; 69: 875–899. 11777114

[bib27] Juster HR, Heimberg RG, Engelberg B. Self selection and sample selection in a treatment study of social phobia. Behav Res Ther 1995; 33: 321–324. 7726809 10.1016/0005-7967(94)e0027-g

[bib28] Lincoln TM, Rief W, Hahlweg K, Frank M, von Witzleben I, Schroeder B et al. Effectiveness of an empirically supported treatment for social phobia in the field. Behav Res Ther 2003; 41: 1251–1269. 14527526 10.1016/s0005-7967(03)00038-x

[bib29] Franklin CL, Zimmerman M. Posttraumatic stress disorder and major depressive disorder: investigating the role of overlapping symptoms in diagnostic comorbidity. J Nerv Ment Dis 2001; 189: 548–551. 11531207 10.1097/00005053-200108000-00008

[bib30] Mueser KT, Rosenberg SD, Xie H, Jankowski MK, Bolton EE, Lu W et al. A randomized controlled trial of cognitive-behavioral treatment for posttraumatic stress disorder in severe mental illness. J Consult Clin Psychol 2008; 76: 259–271. 18377122 10.1037/0022-006X.76.2.259 PMC3916092

[bib31] Gandhi M, Ameli N, Bacchetti P, Sharp GB, French AL, Young M et al. Eligibility criteria for HIV clinical trials and generalizability of results: the gap between published reports and study protocols. Aids 2005; 19: 1885–1896. 16227797 10.1097/01.aids.0000189866.67182.f7

[bib32] Gross CP, Mallory R, Heiat A, Krumholz HM. Reporting the recruitment process in clinical trials: who are these patients and how did they get there? Ann Intern Med 2002; 137: 10–16. 12093240 10.7326/0003-4819-137-1-200207020-00007

