# Supplementary material for: A trans-diagnostic review of anxiety disorder comorbidity and the impact of multiple exclusion criteria on studying clinical outcomes in anxiety disorders
Source: Transl Psychiatry. 2016 Jun 28;6(6):e847–. doi: 10.1038/tp.2016.108 (PMC4931606; doi:10.1038/tp.2016.108)
Supplement: Supplementary Information [file tp2016108x1.doc]

**Supplemental Materials**

eMethods 2

eResults 3

eTable 1 7

eTable 2 8

References 9

**eMethods**

In the Cross-Disease Review of Exclusion Across Medicine (CREAM) project, literature was identified systematically by conducting English-language searches in PubMed (Date of Search: Oct 1, 2014 on the following terms: “Eligibility criteria and generalizability” (anywhere in paper), “exclusion criteria and generalizability” (anywhere in paper), “exclusion criteria” (in title of paper) and “eligibility criteria” (in title of paper). To be considered relevant, studies had to analyze data on i) the prevalence and nature of exclusion criteria, ii) the overall and specific rates of exclusion due to commonly used exclusion criteria, and/or iii) the impact of exclusion criteria on sample representativeness or outcomes. From this cross-disease pool of literature, evidence on individual diseases was extracted for focused reviews, in this case studies addressing anxiety disorders.

**eResults**

**i) Prevalence of exclusion criteria by anxiety diagnosis**

Post-traumatic stress disorder (PTSD). Number of studies on pharmacotherapy/psychotherapy: In 2005, Bradley et al.^1^ conducted a meta-analysis for psychotherapy treatments for PTSD spanning from 1980 through 2003 and included 26 empirical papers. In 2014, Ronconi et al.^2^ conducted a meta-analysis spanning from 1980 through 2012, which included 63 empirical papers (17 overlapped with Bradley et al.^1^), which included 75 separate studies; these included an additional 41 publications conducted between 2003 in 2012, after those in the Bradley et al. report. Despite the importance of exclusion criteria and the information they provide about the generalizability of randomized controlled trial (RCT) findings, Bradley et al. found information missing in nearly 40% of the articles identified (this statistic was not available for Ronconi et al.) See Table 2 for the most common exclusion criteria and the eResults for more detail. Although there were high numbers of exclusion criteria overall, Ronconi et al. noted that no single exclusion criterion was used across all studies, suggesting that PTSD studies overall enroll a representative range of patients even though individual studies often do not.

PTSD. Most common exclusion criteria: Psychosis was the most commonly reported exclusion criteria, appearing in 85% of the studies identified by Bradley et al.^1^ and 91% identified by Ronconi et al.^2^ Other commonly reported exclusion criteria were organic disorders (77% of the studies identified in Bradley et al., percentage not available for Ronconi et al.), substance use (i.e. alcohol and/or drug use included) disorder (62% Bradley et al.; Ronconi et al. further divided this criteria into abuse and dependence, finding 44% and 72%, respectively, included these criteria), and suicide risk (46% Bradley et al.; 59% Ronconi et al.) Other comorbidities (e.g., depression, bipolar disorder) and changes in psychotropic medication were also highly prevalent.

Obsessive-compulsive disorder (OCD). Number of studies on pharmacotherapy/psychotherapy: In 2004, Eddy et al.^3^ conducted separate meta-analyses for psychotherapy and pharmacotherapy OCD treatments, identifying 15 and 32 independent studies, respectively, conducted between 1980 and 2001. Of these, 20% of psychotherapy and 19% of pharmacotherapy studies did not include information regarding exclusion criteria. In 2014, Odlaug et al.^4^ conducted a literature review of RCTs for pharmacotherapy OCD treatments spanning 1980 through 2010 as part of a separate empirical paper (discussed further below), which identified 39 independent studies. However, they did not report the number of studies that used each of the listed exclusion criteria. The pharmacotherapy trials were more restrictive than the psychotherapy trials, having more independent exclusion criteria (12 vs. 8, respectively) and a higher percentage of studies using each individual criteria (range of prevalence across studies: psychotherapy 7%-60%, pharmacotherapy 15%-72%). See Table 2 for the most common exclusion criteria and the eResults for more details. Interestingly, their findings suggest that the number of exclusion criteria used in RCTs has increased over time. They identified three common exclusion criteria from the 9 trials conducted from 1980-1989 compared to 9 independent exclusion criteria from the trials in 1990-2010.^4^

OCD. Most common exclusion criteria: In Eddy et al.,^3^ when collapsing across both types of RCTs, concurrent psychotherapy or pharmacotherapy was the most commonly used exclusion criterion (66% of studies), followed by substance use disorder (62%), psychotic or organic disorder (60%), and major medical conditions (55%). Comorbidity across several Axis I disorders was also commonly used, including depression (47% of studies), agoraphobia (39%), panic disorder (34%), eating disorders (39%), and bipolar disorder (38%). Axis II comorbidity was also common (13%).

Supporting and extending these findings, the study similarly found that among the most commonly used exclusion criteria were lifetime bipolar disorder, lifetime psychotic disorders, or schizophrenia, diagnosis of alcohol or drug abuse or dependence within the last six months, as well as current MDD, comorbid anxiety disorder (other than OCD), and suicide risk. Other highly prevalent exclusions were duration of illness, significant presence of depression symptoms (but not necessarily MDD comorbidity), and inadequate OCD symptom severity.

Panic disorder (PD). Number of studies on pharmacotherapy/psychotherapy: To date, one study has examined the frequency of exclusion criteria usage for PD studies.^5^ It identified 11 of the most commonly used exclusion criteria used in 20 PD pharmacotherapy and psychotherapy trials conducted from 1980 through 2004, all of which provided exclusion criteria. See Table 2 for the most common exclusion criteria.

PD. Most common exclusion criteria: The Hoertel et al.^5^ study found the usage of 1-10 independent exclusion criteria (median: 6). The most common was psychosis (80% of studies), followed by current depression (75%), substance use disorder (60%), and significant medical condition (55%).

Social phobia (SO). Number of studies on pharmacotherapy/psychotherapy: Two reports examined the prevalence of exclusion criteria for SO. In 2004, Lincoln et al.^6^ focused exclusively on cognitive and/or behavioral RCTs completed between 1996 and 2002. They reviewed a total of 30 independent publications, 26 of which had complete information regarding exclusion criteria. In 2014, a report by Hoertel et al.^7^ included a review of exclusion criteria prevalence for both psychotherapy and pharmacotherapy SO treatments. However, the findings are limited to a listing of the most commonly used.

In the Lincoln et al. meta-analysis of psychotherapy RCTs, the individual studies ranged from 0-5 independent criteria. However, this estimate may be low since several of the exclusion criteria are compounded (e.g., exclusion of comorbid psychosis, substance misuse or bipolar disorder are grouped under 1 criteria). These exclusion criteria were also identified in the Hoertel et al. analysis of both pharmacological and psychotherapy RCTs. The median number of exclusion criteria for the 27 pharmacological studies and the 27 psychotherapy studies was 10 and 7, respectively. See Table 2 for the most common exclusion criteria with Hoertel et al. pharmacological and psychotherapy reports shown ranked from those that excluded the largest percentage of patients to those that excluded the smallest.

SO. Most common exclusion criteria: In Lincoln et al.,^6^ the most commonly reported exclusion criteria was comorbid psychosis, substance misuse or bipolar disorder (88% of studies), followed by comorbid depression (62% of studies). These percentages reflect the compound effects of excluding by at least one comorbid Axis 1 psychiatric disorder, with the effect that if one disorder is excluded often other additional comorbid psychiatric disorders are also excluded.

Generalized anxiety disorder (GAD). Number of studies on pharmacotherapy/psychotherapy: To date, one study has examined the frequency of exclusion criteria usage for GAD for both pharmacotherapy and psychotherapy.^8^ However, the findings are limited to listing the most commonly used without providing a distribution across the included studies. The median number of exclusion criteria for the 24 pharmacological studies and the 12 psychotherapy studies was 11 and 5, respectively. See Table 2 for the most common exclusion criteria in pharmacological and psychotherapy RCTs, shown ranked from those excluding the largest percentage of patients to those that excluded the smallest.

***ii) Rates of exclusion***

PTSD. In 2005, Bradley et al.^1^ conducted a retrospective meta-analysis to examine the exclusion rates of participants from previously published RCTs and found that approximately 30% of treatment-seeking patients were screened out. No study to date has examined the degree to which these exclusion criteria apply to treatment-seeking populations of PTSD patients. However, it is important to note that the rates for comorbidities commonly excluded from RCTs—including depression, other anxiety disorders, and alcohol and drug abuse/dependence—are high in PTSD with 86% of individuals with a lifetime history of PTSD experiencing at least 1 other comorbid disorder.^9^

OCD. Of the three studies that evaluated the extent to which exclusion criteria applied to a population of treatment-seeking patients with OCD, two focused exclusively on pharmacotherapy^4,10^ and one examined pharmacotherapy and psychotherapy exclusion criteria separately.^3^

Franklin et al. reported exclusion criteria for a sample of 110 adult outpatients seeking treatment for OCD.^10^ Of these, 79% were deemed ineligible for participation in a pharmacotherapy RCT. The reasons included too low severity of symptoms (4%), comorbid Axis I diagnosis (47%), previous exposure therapy (7%) or pharmacotherapy treatment of the relevant drug (24%), and out of town residence (13%). Focusing specifically on comorbidity rates, they found that 26% of the sample experienced comorbid MDD, an additional 17% had a comorbid Axis II disorder, 12% had a comorbid anxiety disorder, 3% had comorbid bipolar disorder, and 1% had comorbid psychosis.

In a more recent study, Eddy et al.^3^ conducted two meta-analyses on commonly used exclusion criteria for psychosocial and pharmacotherapy RCTs. Of the psychosocial treatments, only 3 of the 15 reviewed studies provided information regarding the degree to which participants were screened out. From these three studies, they determined that an average of 52·62% of patients were excluded (range: 34-85%). Regarding pharmacotherapy RCTs, only 6 of 32 RCTs provided the number and percentage of potential patients excluded. Somewhat surprisingly, despite having a larger number of potential exclusions, the impact of exclusion criteria on those seeking pharmacotherapy RCTs averaged only 33% of patients (range: 3-50%).

In the most recent study, Odlaug et al.^4^ conducted a meta-analysis of pharmacotherapy clinical trials conducted between 1980 and 2010 and derived lists of exclusion criteria that appeared in over 65% of the studies across each decade. They then applied these exclusion criteria to a group of 325 treatment-seeking patients diagnosed with OCD (Table 3). Several relevant findings are of note. First, in terms of general rate of exclusion between 1980 and 2010, 72% of the patients would have been excluded due to meeting at least one exclusion criterion, with 54·2% experiencing a comorbid condition such as another anxiety disorder (42·5%), MDD (16·3%), psychosis (2·8%), alcohol or drug abuse (5·2%), and bipolar disorder (3·1%). Second, they provided evidence suggesting that exclusion criteria have become more restrictive over time. For example, the three exclusion criteria most commonly used between 1980 and 1989 would have excluded only 19·7% of the patients; however, both the number of exclusion criteria and the impact of these criteria increased such that the nine exclusion criteria used in over 65% of the studies between 2000 and 2010 excluded as many as 76·9% of patients.

PD. In 2001, Westen et al.^11^ conducted the first meta-analysis of exclusion criteria in PD. Although they did not identify the impact of specific exclusion criteria on the number of participants screened out, they provided statistics regarding overall exclusion rates. Of the 17 studies analyzed, only seven provided statistics regarding the percentage of patients excluded. From these, they estimated that upwards of 64% of participants were screened out. They claimed that the prototypical exclusion criteria included moderate to severe agoraphobia, any concurrent Axis I or Axis II disorder in need of immediate treatment, primary MDD diagnosis. and previous therapy. Criteria reported less commonly were suicidality or substance abuse.

In 2013, Hoertel et al.^5^ conducted a meta-analysis and derived the six most commonly used exclusion criteria for pharmacotherapy and psychotherapy clinical trials. They then applied these exclusion criteria to an independent population of treatment-seeking individuals diagnosed with PD to determine the extent of exclusion (Table 3). Strikingly, they found that on average, 92·4% would have been excluded by at least one criterion. In fact, having current depression alone was enough to exclude 70·6%, with an additional 61·5% being excluded for current/past 6 months of any substance abuse/dependence and 58·9% for having a comorbid bipolar disorder diagnosis. Further, a significant medical condition and psychosis applied to 34·3% and 21·2% of the population, respectively.

Mavissakalian and Guo^12^ reported the extent of exclusion for 333 adults with panic and agoraphobic-like symptoms who were attempting to enroll in a long-term imipramine treatment study. Of these, 161 participants (48·3% of the total sample) were deemed ineligible for reasons including too low severity of symptoms (83 participants), current or past diagnosis of depression (28 participants), current substance abuse (three participants), current medications (19 participants), health reasons (20 participants), because behavioral treatment was deemed clinically necessary (six participants), patient planned to become pregnant (one participant), or duration of illness too short (one participant).

GAD. In 2001, Westen et al.^11^ conducted the first meta-analysis of exclusion criteria in GAD. Although they did not identify the impact of specific exclusion criteria on the number of participants screened out, they provided statistics regarding the overall exclusion rates. Of the five studies included, only three provided statistics regarding the percentage of patients excluded. From these, they estimated that upwards of 65% of participants were screened out. They claimed that the prototypical exclusion criteria included major depression, substance use disorders, and suicide risk. Criteria reported less commonly were dysthymia, somatic disorders, panic disorders, past psychosocial treatment, current substance abuse, or OCD.

In 2012, Hoertel et al.^8^ conducted a meta-analysis of clinical trials and derived a list of the 11 and five most commonly used exclusion criteria for pharmacotherapy and psychotherapy clinical trials, respectively. They then applied these sets of exclusion criteria to an independent population of treatment-seeking individuals diagnosed with GAD to determine the extent of exclusion. On average, 81·8% were excluded when using the more extensive exclusion criteria derived from the pharmacotherapy trials. This number may underestimate the total as information on three of the most commonly used exclusion criteria (current psychotropic medication, currently receiving psychotherapy, and comorbid OCD) was not available for this population. A current diagnosis of depression excluded the most participants (62·75%) (Table 3).

Similarly, 82·1% of treatment-seeking individuals were excluded when using exclusion criteria derived from psychotherapy trials (Table 3). Specific exclusion rates were available for 4 of the 5 commonly used criteria, the most common being current depression (Table 3). Again, it is important to note that the overall estimate of exclusion rate is conservative because data were not available for current use of psychotropic medication.

SO. In 2014, Hoertel et al. conducted a meta-analysis of clinical trials and derived the 10 and six most commonly used exclusion criteria for pharmacotherapy and psychotherapy clinical trials, respectively.^7^ They then applied these sets of exclusion criteria to an independent population of treatment-seeking individuals diagnosed with SO to determine the extent of exclusion. On average, 87·8% were excluded when using the more extensive exclusion criteria derived from the pharmacotherapy trials. Again, this estimate is conservative because information on current psychotropic medication was not available for this population. A current diagnosis of depression excluded the most participants (60·42%), followed by lifetime bipolar disorder (42·63%) (Table 3).

Similarly, 80·5% were excluded when using exclusion criteria derived from psychotherapy trials. Specific exclusion rates were available for five of the six commonly used criteria, the most common being current depression, followed by lifetime bipolar disorder (Table 3).

**eTable 1. Treatments for anxiety disorder**

| **Type of treatment** | **Examples** | **Used for** |
| --- | --- | --- |
| **Pharmacotherapy** |  |  |
| SSRI (selective serotonin reuptake inhibitor) | Fluoxetine, fluvoxamine, sertraline, paroxetine, escitalopram, citalopram | OCD, PD, SO, GAD, PTSD |
| SNRI (serotonin-norepinephrine reuptake inhibitor) | venlafaxine, duloxetine | OCD, PD, SO, GAD, PTSD |
| Tricyclic antidepressant | amitriptyline, imipramine, nortriptyline , doxepin, clomipramine | PD, GAD, PTSD |
| MAOIs (Monoamine Oxidase Inhibitors). | Phenelzine, tranylcypromine | OCD, PD, SO, GAD, PTSD |
| Other antidepressants | trazodone | GAD |
| Benzodiazepine | alprazolam, clonazepam, diazepam, lorazepam | OCD, PD, SO, phobias |
| Beta-blockers | Propranolol, atenolol | SO |
| Mild tranquilizer | Buspirone | OCD, PD, GAD |
| Anticonvulants | Valproate, pregabalin, gabapentin | PD, SO, GAD |
| **Psychotherapy** |  |  |
| Cognitive Behavioral Therapy |  | OCD, PD, SO, GAD, PTSD |
| Exposure Therapy |  | PTSD |

- Pharmacotherapy and Psychotherapy are also used in combination.

*Abbreviations*: GAD, Generalized anxiety disorder; OCD, Obsessive-Compulsive Disorder; PD, Panic disorder; PTSD, Post-traumatic stress disorder; SO, Social phobia

**e**Table 2: Lifetime prevalence by diagnosis and sex

| **Diagnosis** | **Total Sample** | | **Females** | | **Males** | |
| --- | --- | --- | --- | --- | --- | --- |
|  | **N=9282** | | **N=5143** | | **N=4139** | |
|  | **n** | **%** | **n** | **%** | **n** | **%** |
| Any Anxiety Disorder | 2611 | 33·17 | 1706 | 33·17 | 905 | 21·87 |
| PD | 455 | 4·90 | 320 | 6·22 | 135 | 3·26 |
| PTSD | 604 | 6·51 | 468 | 9·10 | 136 | 3·29 |
| GAD | 752 | 8·10 | 531 | 10·32 | 221 | 5·34 |
| SO | 1143 | 12·31 | 693 | 13·47 | 450 | 10·87 |
| SP | 1198 | 12·91 | 826 | 16·06 | 372 | 8·99 |
| MDD | 1829 | 19·70 | 1212 | 23·57 | 617 | 14·91 |

*Abbreviations*: GAD, Generalized anxiety disorder; MDD, Major depressive disorder; OCD, Obsessive-Compulsive Disorder; PD, Panic disorder; PTSD, Post-traumatic stress disorder; SO, Social phobia

**References**

1. Bradley R, Greene J, Russ E, Dutra L, Westen D. A multidimensional meta-analysis of psychotherapy for PTSD. *Am J Psychiatry* 2005; **162(2):** 214–27.

2. Ronconi JM, Shiner B, Watts BV. Inclusion and exclusion criteria in randomized controlled trials of psychotherapy for PTSD. *J Psychiatr Pract* 2014; **20(1):** 25–37.

3. Eddy KT, Dutra L, Bradley R, Westen D. A multidimensional meta-analysis of psychotherapy and pharmacotherapy for obsessive-compulsive disorder. *Clin Psychol Rev* 2004; **24(8):** 1011–30.

4. Odlaug BL, Weinhandl E, Mancebo MC, et al. Excluding the typical patient: thirty years of pharmacotherapy efficacy trials for obsessive-compulsive disorder. *Ann Clin Psychiatry* 2014; **26(1):** 39–46.

5. Hoertel N, Le Strat Y, De Maricourt P, Limosin F, Dubertret C. Are subjects in treatment trials of panic disorder representative of patients in routine clinical practice? Results from a national sample. *J Affect Disord* 2013; **146(3):** 383–9.

6. Lincoln TM, Rief W. How much do sample characteristics affect the effect size? An investigation of studies testing the treatment effects for social phobia. *J Anxiety Disord* 2004; **18(4):** 515–29.

7. Hoertel N, de Maricourt P, Katz J, et al. Are participants in pharmacological and psychotherapy treatment trials for social anxiety disorder representative of patients in real–life settings? *J Clin Psychopharmacol* 2014; **34(6):** 697–703.

8. Hoertel N, Le Strat Y, Blanco C, Lavaud P, Dubertret C. Generalizability of clinical trial results for generalized anxiety disorder to community samples. *Depress Anxiety* 2012; **29(7):** 614–20.

9. Kessler RC, Berglund P, Demler O, Jin R, Merikangas KR, Walters EE. Lifetime prevalence and age-of-onset distributions of DSM-IV disorders in the National Comorbidity Survey Replication. *Arch Gen Psychiatry* 2005; **62(6):** 593–602.

10. Franklin ME, Abramowitz JS, Kozak MJ, Levitt JT, Foa EB. Effectiveness of exposure and ritual prevention for obsessive-compulsive disorder: randomized compared with nonrandomized samples. *J Consult Clin Psychol* 2000; **68(4):** 594–602.

11. Westen D, Morrison K. A multidimensional meta-analysis of treatments for depression, panic, and generalized anxiety disorder: an empirical examination of the status of empirically supported therapies. *J Consult Clin Psychol* 2001; **69(6):** 875–99.

12. Mavissakalian MR, Guo S. Predictors of entering a long-term drug treatment study of panic disorder. *Compr Psychiatry* 2002; **43(2):** 88–94.
